# Associations between whole peripheral blood fatty acids and DNA methylation in humans

**DOI:** 10.1038/srep25867

**Published:** 2016-05-16

**Authors:** Carmen de la Rocha, J. Eduardo Pérez-Mojica, Silvia Zenteno-De León, Braulio Cervantes-Paz, Fabiola E. Tristán-Flores, Dalia Rodríguez-Ríos, Jorge Molina-Torres, Enrique Ramírez-Chávez, Yolanda Alvarado-Caudillo, F. Javier Carmona, Manel Esteller, Rosaura Hernández-Rivas, Katarzyna Wrobel, Kazimierz Wrobel, Silvio Zaina, Gertrud Lund

**Affiliations:** 1 Department of Genetic Engineering, Center for Research and Advanced Studies of the National Polytechnic Institute (CINVESTAV) Irapuato Unit, 36821 Irapuato, Gto., Mexico; 2 Department of Molecular Biomedicine, CINVESTAV Campus Zacatenco, Mexico D.F., Mexico; 3 Department of Biochemistry and Biotechnology, CINVESTAV Irapuato Unit, 36821 Irapuato, Gto., Mexico; 4 Department of Medical Sciences, Division of Health Sciences, León Campus, University of Guanajuato, Mexico; 5 Cancer Epigenetics and Biology Program (PEBC), Bellvitge Biomedical Research Institute (IDIBELL), L’Hospitalet de Llobregat, Barcelona, Catalonia, Spain; 6 Department of Chemistry, Division of Natural and Exact Sciences, Guanajuato Campus, University of Guanajuato, Mexico

## Abstract

Fatty acids (FA) modify DNA methylation *in vitro*, but limited information is available on whether corresponding associations exist *in vivo* and reflect any short-term effect of the diet. Associations between global DNA methylation and FAs were sought in blood from lactating infants (LI; n = 49) and adult males (AMM; n = 12) equally distributed across the three conventional BMI classes. AMM provided multiple samples at 2-hour intervals during 8 hours after either a single Western diet-representative meal (post-prandial samples) or no meal (fasting samples). Lipid/glucose profile, *HDAC4* promoter and *PDK4* 5’UTR methylation were determined in AMM. Multiple regression analysis revealed that global (in LI) and both global and *PDK4*-specific DNA methylation (in AMM) were positively associated with eicosapentaenoic and arachidonic acid. *HDAC4* methylation was inversely associated with arachidonic acid post-prandially in AMM. Global DNA methylation did not show any defined within-day pattern that would suggest a short-term response to the diet. Nonetheless, global DNA methylation was higher in normal weight subjects both post-prandially and in fasting and coincided with higher polyunsaturated relative to monounsaturated and saturated FAs. We show for the first time strong associations of DNA methylation with specific FAs in two human cohorts of distinct age, diet and postnatal development stage.

The consumption of high-fat diets and alterations in lipid metabolism are associated with metabolic diseases and cancer^1^,^2^,^3^. *In vitro* data indicate that very low density lipoproteins (VLDL) and specific fatty acids (FAs) can change DNA methylation patterns^4^,^5^,^6^,^7^. These data, together with the known association of global DNA hypomethylation with cancer (reviewed by Kulis and Esteller^8^), metabolic syndrome^9^ and the post-rupture atherosclerotic lesion^10^, and of DNA hypermethylation with early-stage atherosclerosis^11^,^12^, suggest that dietary lipids may exert pathological effects at least in part by imposing pathological DNA methylation profiles. If so, associations should be detectable between DNA methylation and specific FAs, yet the topic has received little attention to date.

Another pending issue is whether such associations reflect short- or long-term responses to the diet. Mid- and long-term high-fat dietary supplementation can change DNA methylation profiles in mammals^13^,^14^. Epigenetic short-term effects of the diet could be equally relevant, as the frequent transitions from pre-prandial/fasting to post-prandial states that are experienced by humans, may result in corresponding frequent oscillations between fasting- and post-prandial-specific epigenetic marks in at least selected loci in the genome. If so, it is conceivable that any error-prone diet-driven oscillations may lock selected loci in a pathogenic chromatin state. For example, locking in a fasting-specific state may exacerbate a pre-existing thrifty genotype^15^.

In the present work, we assessed whether any association exists between whole peripheral blood DNA methylation and specific FAs and lipids in two distinct human cohorts - lactating infants and adult men - and whether those associations are rapidly modified in the postprandial state.

## Results

### Associations between DNA methylation and FAs in the lactating infant (LI) cohort

We first asked whether any association existed between global DNA methylation and specific FAs in whole peripheral blood. To that end, we interrogated a cohort of 49 Mexican lactating infants of both sexes. A description of the lactating infant cohort is presented in Supplementary Table 1. Sex, age at sampling, weight at birth, normalized weight gain - *i.e*. difference between the weight at sampling and at birth divided by the birth-to-sampling time (days) - whole peripheral blood FAs and global DNA methylation were the available variables for that cohort. When grouped by degree of saturation and percent-normalized, FAs showed a non-significant tendency to associate inversely (saturated and monounsaturated FAs or SFAs and MUFAs, respectively) or positively (polyunsaturated FAs or PUFAs) with DNA methylation (Supplementary Table 2.1). A multiple regression analysis adjusting for the above mentioned variables only revealed significant positive associations of global DNA methylation with normalized (% of all FAs) n-3 PUFA eicosapentaenoic acid (EPA; C20:5) and the n-6 PUFA arachidonic acid (AA; C20:4) among all detected FAs, with a noticeably stronger (higher Beta) association with EPA (Table 1). Although the estimation of the effects was not the primary aim of the study, the adjusted R^2^ indicates that EPA and AA explain ~17% of the variation in DNA methylation in the presented model. Furthermore, although DNA methylation changes with age^16^, we failed to detected any corresponding association between these two parameters in the LI cohort (Supplementary Table 2.1). However, a handful of FAs were associated with age but not with sex (Supplementary Table 3). Notably, age accounted for nearly half of the variability of the sum of the SFA and MUFA C22:0 and C14:1 (p = 0.0015, adjusted R^2^ = 0.49) and the latter FAs were not associated with DNA methylation (p = 0.184, adjusted R^2^ = 0.04).

### DNA methylation trend across the sampling period in adult men (AMM)

The observation that global DNA methylation was associated with specific FAs in LI, prompted us to ask whether those associations were reproducible in an entirely distinct cohort and whether they were dynamically responsive to the diet. These questions were addressed in the AMM subjects, for which a complete metabolic and anthropometric profile was obtained, in addition to global and gene-specific DNA methylation data.

**Figure 1:**
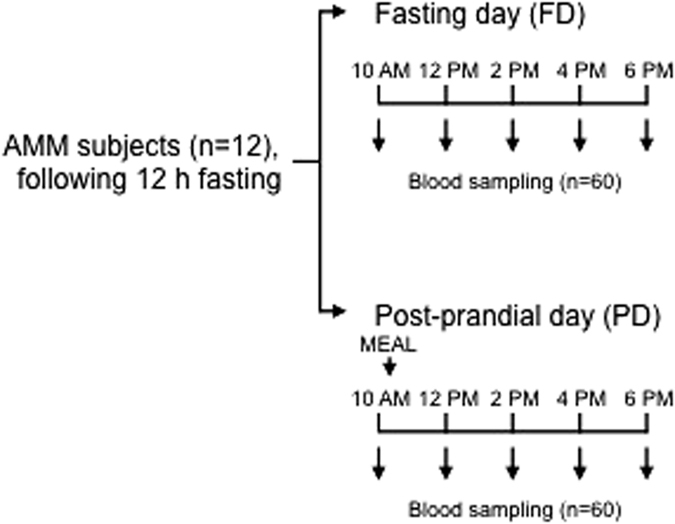
AMM subject sampling scheme. Five consecutive, same-day blood samples were obtained from a total of 12 subjects in two prandial conditions: fasting (FD) and post-prandial (PD). The 10 AM blood sample represented the baseline for each day. A total of 120 samples were obtained and analyzed (60 per prandial condition).

A total of 12 men entered and completed the study. A description of the AMM subjects is presented in Supplementary Table 4. The AMM subjects underwent a repeated blood draw procedure across an 8-hour period (see Methods), which is schematically shown in Fig. 1. Four subjects represented each of the normal weight, overweight and obese groups (BMI < 25, 25–30 and >30, respectively). The comparison between BMI groups showed expected significant differences in body weight, waist and hip circumference (WC and HC, respectively), waist-to-height ratio (WHtR) and systolic blood pressure (SBP). Meanwhile, age, height, waist-to-hip ratio (WHR) and diastolic blood pressure (DPB) showed no differences (Supplementary Table 4).

**Figure 2:**
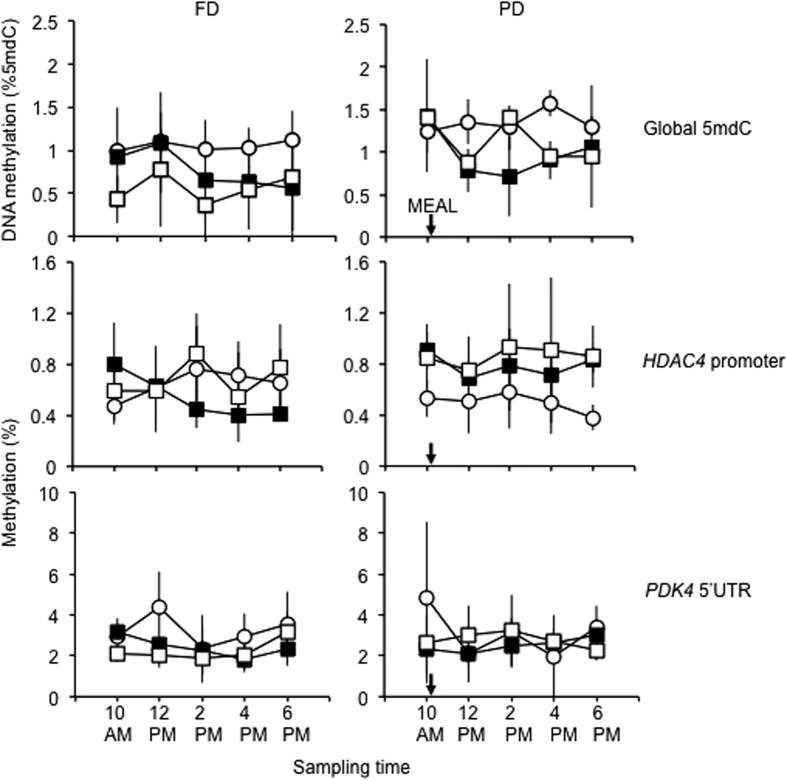
DNA methylation trends across the sampling period in AMM. Average and SD for n = 4 subjects per time point are shown. Open circles: normal weight; solid squares: overweight; open squares: obese. The arrows indicate the approximate time of the meal consumption in PD.

We hypothesized that total blood FA-dependent and/or lipid-dependent trends in global DNA methylation - *i.e*. an increase or decrease relative to 10 AM sample baseline, whether transient or stable throughout the 8 h sampling period - would be observed. However, a highly variable and not identifiable DNA methylation pattern across the sampling period was observed in the PD or FD sets - *i.e*. no significant difference between the 10AM baseline and any other time point or between the maximum and minimum values within any of the 6 sets (3 PD, 3 FD) could be detected (ANOVA and Scheffé's post hoc test; Fig. 2 upper panels). Noticeably, the variability (SD) in the 5 average values (one for each time point) of the normal weight subjects was 3–5-fold lower compared to the other two BMI classes in both PD and FD (p < 0.001 in all comparisons with the normal weight set; Bartlett’s test).

### Associations between DNA methylation and metabolic parameters in AMM

**Figure 3:**
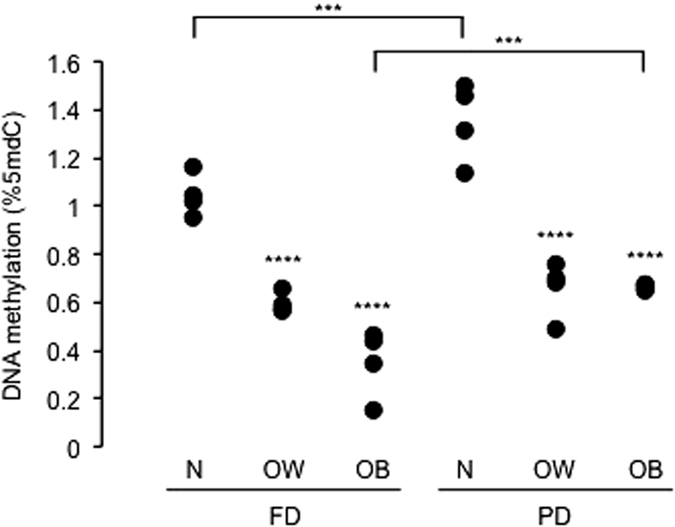
Global DNA methylation in FD, PD and BMI classes. Datapoints are the four subjects’ averages of 5 repeated measurements in each condition. Asterisks above data points indicate the significance of the comparison with normal weight subjects within the FD or PD set. Asterisks above the horizontal lines indicate the significance of the comparison between FD and PD for the same BMI group. N, OW and OB indicate normal weight, overweight and obese subjects, respectively. Significance levels: ***p < 0.001; ****p < 10^−4^; ANOVA and Scheffé's test.

Based on the lack of any time-dependent global DNA methylation pattern and with the aim of optimising measurement accuracy, we treated the 5 PD or FD time-point data for each AMM subject as repeated measurements. The analysis of such subject-averaged data revealed an interplay between global DNA methylation, post-prandial state and BMI. First, global DNA methylation was significantly higher in PD relative to FD when comparing normal weight or obese AMM of the two sets (Fig. 3). Furthermore, within FD or PD, global DNA methylation was significantly lower in overweight and obese, compared to normal weight subjects. Weak (p = 0.04) differences between overweight and obese subjects were observed and only in the FD set. The extreme global DNA methylation differences and levels shown in Fig. 3 and in the results below, although counterintuitive have been reported in several studies (see for example^17^,^18^,^19^). As for metabolic parameters, an initial approach by Pearson’s correlation analysis revealed that VLDL and TG were significantly and inversely correlated with global DNA methylation in both the FD and PD sets (Supplementary Table 5). To account for a possible delayed response, we compared global DNA methylation at 6 PM against metabolic variables at the 12 PM blood draw, but correlations were generally weaker (Supplementary Table 6).

**Figure 4:**
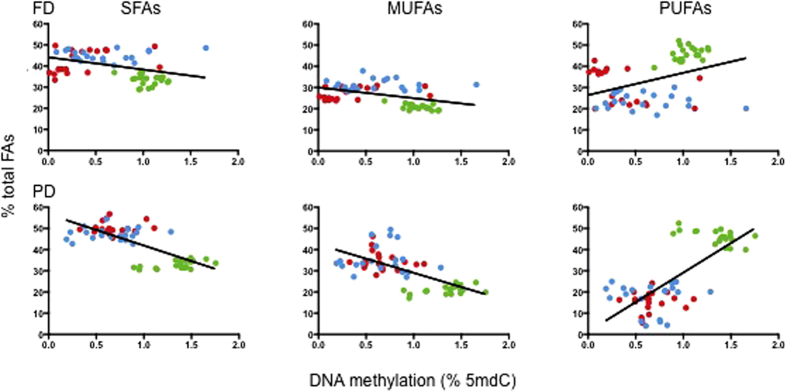
FA type distribution according to BMI and global DNA methylation in AMM. Green, blue and red: normal weight, overweight and obese subjects. n = 20 datapoints for each BMI class are shown, corresponding to the 5 repeated measurements for each of the 4 subjects in each BMI class.

Although the correlation with total FAs was not significant, the above results suggested that specific components of the VLDL FA pool could be associated with global DNA methylation. To explore the potential interplay between FAs and DNA methylation, we examined the distribution of FAs across sample groups, according to the degree of FA saturation. In order to take the data variability into account, we analysed the complete data point set (n = 5 in each time point) for each subject in either FD or PD, *i.e*. n = 20 observations per BMI group. Normalized (% of total FAs) FA data were used. When plotted by global DNA methylation, a clear stratification of FA types could be observed despite the fact that data were not subject-averaged, which suggested specific associations with both global DNA methylation and BMI (Fig. 4). Both SFAs and MUFAs showed an inverse trend relative to DNA methylation, while the opposite was observed for PUFAs. Concomitantly, the data suggested an increase of SFAs and MUFAs, and a decrease of PUFAs, respectively, with BMI. PUFAs were clearly stratified by BMI, particularly in the PD set.

Based on the above data, we undertook a multiple linear regression analysis to assess the association of DNA methylation with normalized (% of total) FAs while adjusting for anthropometric and metabolic variables. The analysis was performed in subject-averaged values (n = 12 for either FD or PD). Global DNA methylation was significantly associated with PUFAs, but not SFAs or MUFAs, in PD and in FD after adjusting as indicated in the Methods section (Table 2). Multiple linear regression analysis within PUFAs revealed a significant positive association of global DNA methylation with EPA in both FD and PD. A significant although weaker positive association with AA was detected but only in PD. Global DNA methylation was inversely associated with BMI in FD and PD. An additional positive association with glucose was detected in PD. Generally, the multiple regression models, including FAs and metabolic variables, accounted for a higher portion of global DNA methylation variation (60–70%) in AMM compared to LI (compare Table 2 and Table 1).

Akin to the LI cohort, we also tested the associations between FAs and age in AMM. A number of significant positive associations (which included both AA and EPA) were consistent in both FD and PD, but none overlapped with those identified in the LI cohort (Supplementary Table 7).

### Associations with gene-specific DNA methylation

Next, we probed for any trend or associations between FAs and the histone deacetylase 4 (*HDAC4*) promoter or pyruvate dehydrogenase kinase 4 (*PDK4*) 5′UTR methylation across sampling time in the AMM subjects. These particular genes and genic regions were chosen based on previous evidence showing an inverse correlation between expression and the methylation state of these regions^20^,^21^,^22^ (Silva-Martínez *et al*., in press). Furthermore, *HDAC4* and *PDK4* show opposite trends in methylation and expression in obese individuals^20^,^21^,^22^ and *PDK4* was the most up-regulated gene in response to the VLDL-rich lipoprotein-induced DNA hypermethylation in the human monocytic THP-1 cell line^5^.

For *HDAC4*, we analysed average methylation data for 2 CpGs located in the distal promoter region, one of which showed a ~40% decrease in methylation between 1 and 100 μM AA-stimulated human cultured THP-1 monocytes that was associated with an increase in expression (Silva-Martínez *et al*., in press). In the case of *PDK4*, we analysed average methylation data of five CpGs that mapped within the 5′UTR region that shows an inverse association with expression when methylated in both a CG and non-CG context (Supplementary Figure 1)^21^. Importantly, traditional bisulfite sequencing of this region in two AMM subjects in PD and FD (n = 20) demonstrated a concordance between the average of bisulfite-sequenced CpGs (n = 35) or non-CpG (n = 83) site methylation and the average methylation of the five pyrosequenced CpGs (Pearson’s r = 0.72, p = 3.4 × 10^−3^ and r = 0.51, p = 0.022, respectively), indicating that average methylation data of the latter is representative of the 5′UTR region.

The average methylation data of the 2 (*HDAC4*) and 5 (*PDK4*) CpGs sequenced were: 0.62 + 0.17% (FD) and 0.67 + 0.25% (PD) for *HDAC4* and 2.70 + 0.69% (FD) and 3.63 + 2.87% (PD) for *PDK4*. Similarly to global DNA methylation, no significant trend with time could be observed for methylation in either gene (Fig. 2 middle and lower panels). However, with respect to variability, *PDK4* showed the opposite pattern to global DNA methylation, *i.e*. a ~2-fold higher variability in the normal weight set, compared to overweight and obese (p < 0.001 in both FD and PD, Bartlett’s test). Gene-specific differences in methylation levels were also observed. *HDAC4* methylation was lower in normal weight PD compared to overweight and obese (p = 0.0006 and p = 5.94 × 10^−5^, respectively; ANOVA followed by the Scheffé's post hoc test). The opposite trend, *i.e*. higher methylation in the normal weight set, was observed in FD for *PDK4*, although only compared to the obese set (p = 0.047). The paired differences between the same BMI class sample in FD and PD were not significant. The data therefore hinted at inverse and positive relationships of *HDAC4* and *PDK4* methylation, respectively, with PUFAs, given the relative enrichment of the latter in the normal weight set. Indeed, multiple regression analysis revealed that *HDAC4* methylation in PD and *PDK4* methylation in FD was inversely and positively associated with AA, respectively (Tables 3 and 4). *PDK4* methylation showed a positive association with EPA in both FD and PD (Table 4). Similar to global DNA methylation, the association with EPA was stronger than with AA. The FA models accounted for 25–70% of the respective DNA methylation variation. Notably, glucose accounted for ~85% of *HDAC4* methylation variation in FD. Examples of the methylation trend at the two loci in two representative samples with extreme AA levels are shown in Supplementary Figure 1. Despite the obvious stratification by BMI class (Fig. 2), only a marginal association of *HDAC4* methylation with BMI was detected in our multiple regression model in PD (Supplementary Table 2.5).

### Associations between FAs, weight or BMI

In order to better understand the interplay between FAs, DNA methylation and the metabolism, we asked whether any specific associations of FAs with body weight existed in LI or AMM. In LI, three FAs were inversely associated with the weight at birth or the normalized weight gain (Supplementary Table 8). In all cases, the portion of weight variance accounted for by these associations was ~17% or less. In AMM, significant associations were observed for the SFA C16:0 (palmitic acid) and the MUFA C16:1 (palmitoleic acid) and for the SFA C18:0 (stearic acid) in FD (Supplementary Table 9). None of these FAs was significantly associated with global or gene-specific DNA methylation (Tables 1 and 2). In summary, associations with weight involved different FAs in LI, PD and FD, and the same FA (C16:0) showed inconsistent associations between LI and AMM.

## Discussion

Although the majority of DNA methylation profiles which are established during early development, are faithfully inherited following replication, several evidences show that the DNA methylation state of specific genes or repetitive sequences is altered, albeit modestly, in response to metabolic changes such as exercise^23^,^24^,^25^, alterations in body weight following RYGB^22^,^26^,^27^,^28^ and dietary intervention^13^,^14^,^29^,^30^,^31^,^32^,^33^. Similarly, our data show that variation in DNA methylation between two different metabolic states, postprandial and fasting, occurs both in lean, overweight and obese individuals. As for post-prandial time-dependent global or gene-specific DNA methylation trends, our survey did not clearly show any consistently identifiable profile. Thus, the data do not support the occurrence of diet-driven, short-term intra-individual global DNA methylation oscillations in an 8-hour time window. Rather, the significant increase in global DNA methylation in the PD sets at least in the two extreme BMI groups compared to FD, suggests dietary effects that are subtle and diluted over time.

We detect an inverse association between BMI and global DNA methylation, which includes potential methylation changes in high copy number repetitive elements, such as LINE and Alu. Several studies have used the methylation state of such repeated elements to probe for associations between BMI and DNA methylation, but in the majority of cases no significant associations or trend, was uncovered (reviewed by van Dijk an colleagues^34^). Similarly, genome-wide obesity-relevant studies of DNA methylation using methylation arrays show either a net increase^26^ or decrease in methylation^35^,^36^,^37^,^38^ of specific genes in obese relative to lean individuals. While the underlying heterogeneity of these results is not understood, several factors such as gender and race/ethnicity^39^,^40^,^41^,^42^, folate and homocysteine levels^41^,^43^, aging and nutritional intake^44^ are all known to affect methylation. Notwithstanding these variables, our results show that EPA and AA are significantly associated with DNA methylation in two cohorts of distinct diet and post-natal developmental stage, independent of BMI and sex. The consistency and relevance of those associations are further evidenced by the fact that global DNA methylation was measured by two entirely different methods (the MethylFlash™ ELISA-based system and an HPLC-based technique) in different laboratories and sample types - *i.e*. whole blood in LI and PBMCs in AMM. Although we also uncovered associations of specific FAs with age and weight, none were consistent across cohorts or related to DNA methylation. Thus our data do not support the idea that specific fatty acids mediate age-related changes in DNA methylation.

Collectively, our data show that whole blood levels of selected PUFAs, which are lower in obese individuals, are important determinants of both global and gene-specific methylation profiles. To our knowledge this is a little explored topic, the only related report describing an inverse association between PUFAs and BMI in healthy obese individuals^45^. Interestingly, a recently published DNA methylome study in twins revealed that only twins discordant for both BMI and elevated liver fat differed significantly in leukocyte DNA methylation, with the heavier twin showing decreased levels of methylation in addition to lower levels of the PUFA linoleic acid^46^. These data point to an important association between liver lipid metabolism and blood methylation profiles. Indeed, the above discussed discrepancies between BMI and DNA methylation may in part be explained by the observations that elevated liver fat content, a characteristic of non alcoholic dependant fatty liver disease, is present in many, but not all obese individuals and disproportionately in Hispanics, predominantly of Mexican origin^47^.

The present analysis included two genes, *HDAC4* and *PDK4*, both of which are known to be important regulators of glucose and fatty acid metabolism^23^,^26^,^48^. In particular, we found that the opposite associations of *HDAC4* and *PDK4* methylation with PUFAs are consistent with the previously published methylation and expression trend of these two genes with BMI^20^,^22^. However, we have recently shown that AA and the MUFA oleic acid have distinct, wide-spread, albeit small effects on a large number of genes in a monocytic THP-1 cell line, some of which are associated with changes in gene expression (Silva-Martínez *et al*., in press). We predict that some of the target genes identified in the above described study will show BMI-dependant changes in methylation and expression, although the effects may be subtle. In line with this view, DHA and EPA intake Yup’ik Alaskan natives, measured using red blood cell δ15N, identified several biologically relevant CpGs sites that showed an robust association between methylation and DHA and EPA intake, of which the majority showed increased levels of methylation^49^. Furthermore, dietary supplementation with n-3 PUFA in pregnant women is associated with a 1% increase in infant LINE-1 methylation and alterations in promoter levels of immune response genes IFNγ and IL13^30^. Similarly, n-3 PUFA or fish oil supplementation in adult humans also lead to modest alterations in promoter regions of genes involved in PUFA metabolism^31^ or genes previously known to be regulated by n-3 FA, the latter of which included *PDK4*^32^.

The associations uncovered here may reflect a direct effect of FAs on the DNA methylome. In support of this view, our recent work identified PPAR-alpha and SIRT1 as mediators of AA-induced DNA hypermethylation in cultured monocytes (Silva-Martínez *et al*., in press). In addition, functional annotation analysis of the top 150 genes (ranked by the number of differentially methylated CpGs between the 100 and 1 μm AA concentrations/number of probes present in Illumina’s Infinium HumanMethylation450 BeadChip platform for a given gene) revealed a significant enrichment for the G protein-coupled receptor signalling pathway that are known to be activated by specific fatty acids^50^. On the other hand, a complex metabolic network links the availability of methyl group donors, lipid metabolism genes and FA synthesis, in which the hepatic metabolic activity plays a central role (reviewed by da Silva and colleagues)^46^,^51^,^52^. Interestingly, hyperhomocysteinaemia, an imbalance of the folate cycle that often results in DNA hypomethylation, is associated with low levels of AA in mice^53^. These studies suggest that the positive association between PUFAs and DNA methylation reflects a balanced cellular metabolism in which the correct methyl donor availability maintains physiological levels of DNA methylation and the correct expression of AA-generating enzymes such as FADS2. To our knowledge, no information is available on the opposite mechanism, *i.e*. a direct effect of FAs on methyl donor availability.

We also document a complex interplay between BMI, high-fat diet (PD set) and DNA methylation. On the one hand, global DNA methylation decreases with BMI in both PD and FD sets, yet an increase is observed in PD relative to FD. It is tempting to speculate that the data reflect opposite short-term and long-term effects of high fat diet. This view is supported by the documented induction of DNA hypermethylation by short-term high-fat diet in humans and mice, and by a 24- exposure of cultured cells to lipoproteins or selected FAs^4^,^5^,^13^,^54^ (and Silva-Martínez *et al*., in press) whereas a 6 year-long high-fat diet induced the opposite response in leukocytes in a primate model^55^. We speculate that the transient BMI-independent increase in DNA methylation observed in PD relative to FD reflects a genomic response to an acute increase in nutrient-activated pathways relevant to glucose and lipid metabolism such as the described pathways involving G-protein coupled signalling and PPARs. Oppositely, the reduced levels of DNA methylation characteristic of overweight and obese individuals may be related to structural changes in liver morphology and/or lipid metabolism following prolonged hyperlipidaemia.

Both global and gene-specific DNA methylation was more strongly associated with EPA than with AA. Yet, EPA circulating levels were 2–3-fold lower than other PUFAs such as docosahexaenoic acid in our samples, in accordance with previously reported data^56^, thus underlining EPA’s significant biological importance. EPA and AA are products of essential FA metabolism, therefore are potentially relevant to understand the epigenetic effects of dietary components in human health and disease. Both FAs are involved in inflammation resolution^57^. The n-3 PUFA EPA has been long regarded as a protective FA, particularly in the light of the favourable cardiometabolic effects of fish oil (see Kromhout and De Goede for a recent review^58^). A growing literature documents the regulation of specific signalling pathways and gene-specific methylation by EPA *in vitro*^59^,^60^. At chromatin level, EPA decreases the expression of polycomb group proteins that alter histone posttranslational histone modifications at specific loci^61^. As for AA, this PUFA has complex biological functions, ranging from the participation in inflammation resolution mentioned above, to being a precursor of inflammatory mediators. The impact of AA on cardiovascular health and other inflammation-related disorders has not yet been conclusively established^62^. We showed that AA induces global DNA hypermethylation in human THP-1 monocytes (Silva-Martínez *et al*., in press), in accordance with the positive association detected in the present study.

In summary, we demonstrate for the first time a strong association of DNA methylation with EPA and AA in two populations of different age and developmental stage. In addition, this study replicates in human subjects *in vivo* data previously obtained in cell lines, underlining the significance of the latter models to mechanistically link EPA and AA to DNA hypermethylation. Further work is needed to correct important *caveat* in this study, such as the relatively small sample size and missing information on potentially diet-dependent blood cell type-specific DNA methylation profiles.

## Methods

### LI cohort

Infants were recruited at the public paediatric centre “Secretaría de Salud UIMAPS San Felipe de Jesús”, in the city of León, state of Guanajuato, in central Mexico, which provides routine infant health checks. Inclusion criteria were exclusive breast-feeding or sporadic use of formula milk (reported in 4 out of 49 infants), absence of any mother’s or infant’s chronic or acute diseases, normal birth weight, uncomplicated pregnancy and delivery. The requirement for predominant breast-feeding aimed at increasing sample homogeneity. All participating mothers gave their informed consent to the procedure and the ethical committee of the hosting institution approved the protocol, which included a presentation of the main results of the study to the mothers and medical personnel. The methods were carried out in accordance with the approved guidelines. The LI cohort and AMM (see below) procedures were conducted in accordance with the WMA’s 2013 Declaration of Helsinki guidelines.

### AMM group participants

Volunteers were recruited among the caretaker personnel of the University of Guanajuato in central Mexico and selected according to metabolic profiling prior to the project. The inclusion criteria were: male sex; age >25 years; normal fasting glucose and total cholesterol (TC) (<100 mg/dL and <250 mg/dL, respectively); normal blood pressure (BP; range: 90–139/60–89 mmHg). Subjects were grouped according to body mass index (BMI) in normal, overweight and obese (n = 4 in each group). The inclusion of males only was aimed at simplifying the statistical analysis and increasing the chances of detecting any effects in the light of the documented sexual dimorphism of obesity and cardiovascular risk^63^,^64^. All participants gave their informed consent to the procedure and the Ethical Committee of the Department of Medical Sciences, University of Guanajuato, approved the protocol. The methods were carried out in accordance with the approved guidelines.

### Blood samples

In the LI cohort, 500 μL of arm venous blood were drawn in an EDTA-coated tube and immediately frozen. In the AMM group, ~10 mL peripheral blood were obtained in two different days apart by at least one week. On both days, the subject was asked to fast for 12 h and to abstain from exercise in the 48 h period prior to the first blood draw. On either day, venous blood was drawn 5 times at 2 h intervals starting at 10 AM. On one day (post-prandial day; PD data set), the subject consumed a single standard meal representative of the Western diet (one each of McDonald’s McChicken, medium-size fries, medium-size coke) immediately after the first blood draw at 10 AM. The 10 AM blood draw data are therefore considered as basal for that day. On the other day (fasting day; FD dataset), the subject maintained fasting throughout the blood draw period and was offered a small meal after the last (6 PM) blood draw. The rationale for including a complete fasting sample set was that it could allow accounting for circadian rhythm-related^65^ and blood cell type composition-related effects. Blood cell-type composition effects would be attenuated in intra-subject comparisons - *i.e*. between PD and FD - by the similar habits the subjects were required to adhere to in the 48 h previous to sampling. Blood cell-type composition may well contribute to inter-individual differences in FA profiles, global DNA methylation levels or other parameters, but correcting for that variable would mask genuine markers of prandial or metabolic state, whatever their underlying biological origin. Water was allowed *ad libitum* throughout the procedure in all cases. A schematic diagram of the procedure is shown in Fig. 1. Peripheral blood mononuclear cells (PBMCs) were obtained from ~5 mL EDTA-anticoagulated blood by centrifugation in Ficoll-Paque (GE Healthcare Life Sciences) according to the manufacturer’s recommendations. PBMCs and the remaining whole blood samples were stored at −80 °C in 200 μL aliquots until processed.

### Blood lipid and FA determination

Standard enzymatic-colorimetric methods (Spinreact) were used to determine triglycerides (TG), high-density lipoproteins (HDL) and TC levels according to the manufacturer’s instructions. Low-density lipoproteins (LDL) and VLDL levels were determined using the Friedewald equation (LDL = TC-HDL-(TG/5 = VLDL)^66^.

Whole blood total (*i.e*. esterified and free) FAs were determined in LI and AMM with two different methods by two different research groups in different laboratories at two different time points. For AMM, 500 μL whole blood aliquots were frozen-lyophilized. One mL 0.5 M NaOH in methanol and 10 μL methyl-nonadecanoic acid (5 mg/mL) as an internal standard, were added and incubated at 90 °C for 1 h. After cooling to room temperature, 1 mL BF3 in methanol was added and incubated at 90 °C for 30 min and cooled again at room temperature. After transferring to a clean vial, 2 mL deionized water and 4 mL hexane were added, the organic phase was collected and the solvent evaporated with a stream of nitrogen. Dried sample were resuspended in 400 μL isooctane to be analysed by GC/IEMS. Samples were analysed in a Gas Chromatograph (Agilent Technologies 7890A) using a capillary column Zebron ZB-WAX (30 m × 250 μm × 0.25 μm) coupled to an Electron Impact Mass Spectrometer (Agilent Technologies 5975C). Oven temperature program was follows: initial temperature 50 °C during 3 min, then increasing at a rate of 10 °C/min to 250 °C and maintained for 20 min. Injector temperature of 220 °C was maintained constant. Helium 2 ml/min was use as carrier gas. Sample was injected with a 10:1 split. MSchem software was used to collect *m/z* data and NIST MS Search Software to compare the mass spectra and retention time vs. NIST 2011 database (National Institute of Standards and Technology Mass Spectra Database U.S. Department of Commerce) for the identification of the fatty acids methyl esters. For LI, FAs were determined as described above but using gas chromatography with flame ionization detector using a HP-88 capillary column (100 m × 0.25 mm, film 0.20 μm, Agilent Technologies, USA). Methyl tridecanoate was used as the internal standard, five point calibration was performed using a FAMEs standard mix containing esters of C8-C22 FAs (Supelco reference number 18920) with addition of AA. The identification of FAs in blood extracts was based on their retention times.

### Global DNA methylation assays

Whole blood (0.2 mL) or PBMC DNA was extracted by using the DNeasy Blood & Tissue Kit (QIAGEN) according to the manufacturer’s instructions and was quantified using SYBR Green I (Sigma-Aldrich). Total 5-methyldeoxycytosine (5mdC) was determined with the MethylFlash™ Methylated DNA Quantification Kit, colorimetric (Epigentek) according to the manufacturer’s instructions or by a HPLC-based method^67^ for AMM or LI, respectively.

### AA-stimulated human THP-1 monocytes

THP-1 cells were stimulated with 1, 10 or 100 μM BSA-conjugated AA for 24 h as described (Silva-Martínez *et al*., in press). Genome-wide methylation data were obtained with the Infinium HumanMethylation450 BeadChip platform (Illumina) and are publicly available in NCBI’s Gene Expression Omnibus^68^ with the accession number GSE67331.

### Gene-specific DNA methylation and expression

DNA methylation in the 5′UTR of pyruvate dehydrogenase kinase, isozyme 4 (*PDK4*) and the histone deacetylase 4 (*HDAC4*) distal promoter was measured by pyrosequencing in bisulfite-treated DNA (EZ DNA Methylation™, Zymo Research), using the PyroMark Q96 ID (Qiagen) according to the manufacturer’s instructions. The following primers designed with the PyroMark Assay Design software were used for *PDK4* pyrosequencing (5′ to 3′): GGTGGGAAGATTTGAATTTGAA and AAAACTACTCRAAACAAAACCTAATTCC (PCR primers; R indicates A or G); GGTATTTTTAAATTTTAGTTTAGG (sequencing primer). The corresponding primers for *HDAC4* were: TGGGAGGTTTGTGTTGAGTT and CACCACCAAAAAAATAACCACTA (PCR primers); TGGTTGTTAGTAGGTG (sequencing primer). Supplementary Figure 1 schematically shows the sequenced loci and primer positions. For profiling the *PDK4* 5′UTR by traditional bisulfite-modified DNA sequencing, the primers AAATGTTTTATTTTTYGGGGTA (forward; Y indicates C or T) and AACTCCTCCTATTTAAAACT (reverse) were used. Fifteen-twenty clones were sequenced per sample. *HDAC4* expression was assessed by reverse transcription-PCR with the primers GTGGGTTTCAACGTCAACATG and GTGACACGGGAAAGTTTCTTG.

### Statistical analysis

Groups were compared by ANOVA followed by Scheffé’s *post hoc* test, while the Wilcoxon test was used in paired comparisons between experimental days. Correlations were measured with the Pearson’s r test. Associations between DNA methylation and FAs were assessed by best fit multiple regression analysis with global or gene-specific DNA methylation as dependent variable. In the case of the LI cohort, percent-normalized total FAs, sex, age at sampling, weight at birth, birth-to-sampling weight change/day (*i.e*. normalized weight gain) were the predictors. In the case of AMM, predictor variables were age, BMI, lipoproteins, TG, cholesterol, total FAs, percent-normalized individual FAs or grouped by degree of saturation and BP. Multiple regression analysis was then repeated with the individual members of the FA saturation class that was significantly associated in the first analysis and all other significantly associated variables as predictors. Dependent variables for all other multiple regression models are indicated in the Results section. Differences in variability (SD) between groups were tested by the Bartlett’s test. Tests were performed with the STATISTICA software (StatSoft) or with the StatPlus (AnalystSoft) MS Excel plug-in.

## Additional Information

**How to cite this article**: de la Rocha, C. *et al*. Associations between whole peripheral blood fatty acids and DNA methylation in humans. *Sci. Rep*. **6**, 25867; doi: 10.1038/srep25867 (2016).

## Supplementary Material

Supplementary Information

## Figures and Tables

**Table 1:** Significant associations of FAs with global DNA methylation (dependent variable) in the LI cohort.

Variable	Beta	t	p	adjusted R^2^
C20:4 (AA)	0.0848	2.1084	0.0420	0.1683
C20:5 (EPA)	0.9867	2.3512	0.0384	

Beta values indicate the change in % 5mdC per one-point increase in percent FA. Non-significant associations are shown in Supplementary Table 2.1.

**Table 2:** Significant associations with global DNA methylation (dependent variable) in the AMM subjects.

Sample	Variable	Beta	t	p	adjusted R^2^
PD	PUFAs	0.0214	3.7356	0.0047	0.4815¶
	BMI	−0.1611	−3.1200	0.0268	0.7437§
	glucose	0.0849	3.2093	0.0237	
	C20:4 (AA)	0.1118	4.2941	0.0013	
	C20:5 (EPA)	0.7000	6.1371	0.0001	
FD	PUFAs	0.0146	2.5549	0.0309	0.4273¶
	BMI	−0.0862	−2.9481	0.0163	0.6235§
	C20:5 (EPA)	1.1339	4.2449	0.0017	

^¶^SFA/MUFA/PUFA model.

^§^Individual PUFA model. Beta values indicate the change in % 5mdC per one-point increase in FA (%), BMI or glucose (mg/dl). Non-significant associations are shown in Supplementary Table 2.4.

**Table 3:** Significant associations with *HDAC4* promoter methylation in the AMM subjects.

Sample	Variable	Beta	t	p	adjusted R^2^
PD	C20:4 (AA)	−0.0664	−2.0042	0.0464	0.2524
FD	glucose	0.0397	4.7465	0.0177	0.8581

Beta values indicate the change in % 5mdC per one-point increase in percent FA. Non-significant associations are shown in Supplementary Table 2.5.

**Table 4:** Significant associations with *PDK4* 5′UTR methylation (dependent variable) in the AMM subjects.

Sample	Variable	Beta	t	p	adjusted R^2^
PD	C20:5 (EPA)	4.1432	2.2777	0.0499	0.3140
FD	PUFAs	0.0373	2.7347	0.0291	0.6990¶
	C20:4 (AA)	0.2411	2.9776	0.0155	0.5828§
	C20:5 (EPA)	1.6523	2.6578	0.0261	

^¶^SFA/MUFA/PUFA model.

^§^Individual PUFA model. Beta values indicate the change in % 5mdC per one-point increase in percent FA. Non-significant associations are shown in Supplementary Table 2.8.
