# Supplementary material for: Associations between whole peripheral blood fatty acids and DNA methylation in humans
Source: Sci Rep. 2016 May 16;6:25867. doi: 10.1038/srep25867 (PMC4867649; doi:10.1038/srep25867)
Supplement: Supplementary Information [file srep25867-s1.pdf]

Supplementary Material

Manuscript # SREP-15-34573A

Title: Associations between whole peripheral blood fatty acids and DNA methylation in humans

Authors: Carmen de la Rocha, José E. Pérez-Mojica, Silvia Zenteno-De León, Braulio Cervantes-Paz, Fabiola E. Tristán-Flores, Dalia Rodríguez-Ríos, Jorge Molina-Torres, Enrique Ramírez-Chávez, Yolanda Alvarado-Caudillo, F. Javier Carmona, Manel Esteller, Rosaura Hernández-Rivas, Katarzyna Wrobel, Kazimierz Wrobel, Silvio Zaina, Gertrud Lund

Content:

Supplementary Figure 1

Supplementary Tables 1 - 9

A

human *HDAC4* promoter, chromosome 2:239,401,946-239,402,052 (- strand)

-993 *TGGGAGGCTTGTGTTGAGTT*ACAGGTCGGCCCGTAGATCCCCGGAAGCGCCTT

TGGTTGCCAGCAGGTGTCGGC*GAGGGTCTT*CAGTGGCCACCCTCCTGGTGGTGA* -1,100

(CpG 1 = TCG; CpG 2 = GCG, marked *)

B

human *PDK4* 5'UTR, chromosome 7:95,596,341-95,596,522 (- strand)

→

+92 *GGTGGGAAGACTTGAACTTGAA*TCTCGAACCACTGCATCTCCGACTCTGCCCAGA

CTCTTCACTCCGC**GGCACCCTCAAACCCCAGCCCAGG**CCGGGGCGCACAAGCCAGCC

AGCGCACCTGCAGTCCTCGCCCGGACGCGCCGCGCCCCCTCG*GAACCAGGCTCTGCT*

CCGAGCAGCCTTC +273

(CpGs numbered 1–5)

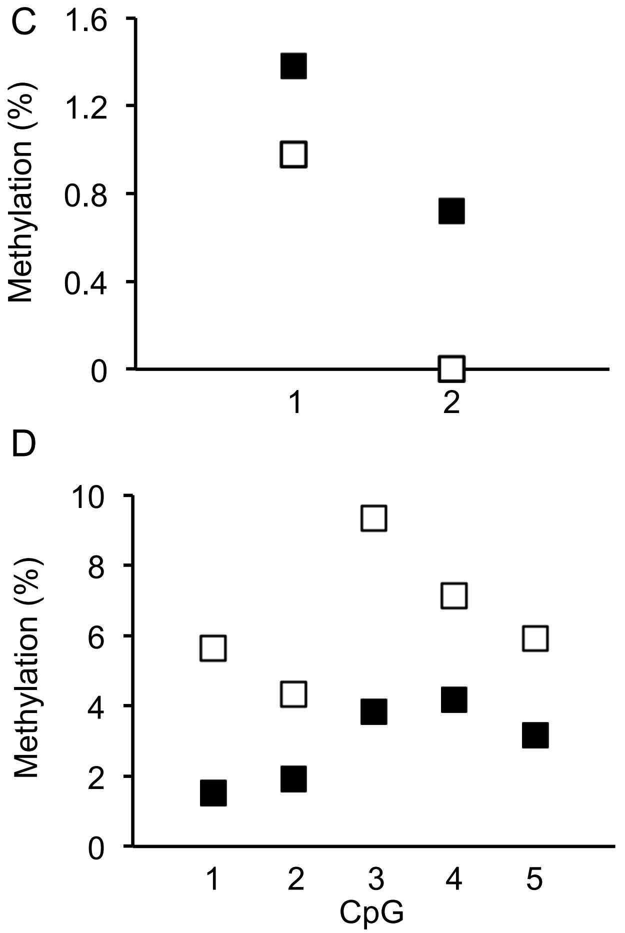

Supplementary Figure 1 – Example of pyrosequencing data for *HDAC4* and *PDK4*. A and B) Position of each sequenced CpG (underlined and numbered) in *HDAC4* and *PDK4* respectively. Priming sites for PCR and sequencing oligonucleotides are in underlined italics and bold, respectively. The asterisk in *HDAC4* marks the CpG that is hypomethylated in response to AA in THP-1 cells (cg15142485 in Illumina's Infinium HumanMethylation450 BeadChip; Silva-Martínez et al., in press). The boxed *PDK4* sequence is the priming site for the 5' oligonucleotide (extending left-to-right) used for bisulfite sequencing by Kulkarni et al. (ref. 21 in main text). The numbers at the sequence ends indicate the distance from the transcription start site (bp). The arrow in *PDK4* indicates the direction of transcription. Coordinates were determined by BLAT using the hg38 assembly. C and D) Methylation levels of individual CpGs (numbered as in A,B) of *HDAC4* and *PDK4*, respectively, in two AMM samples with ~2-fold difference in AA levels – *i.e.* 3.72% (solid squares) and 8.79% (open squares) are shown.

Supplementary Tables

Supplementary Table 1. General characteristics of the LI cohort (n=49).

Females (n)	27
Males (n)	22
Weight at birth (kg)	3.2±0.07 (2.1-4.1)
Weight at sampling (kg)	5.4±1.6 (2.6-8.6)
Age at sampling (days)	89.6±68.2 (10-333)

Continuous variables are represented as average±SEM (minimum-maximum).

Supplementary Table 2 - Non-significant multiple regression results.

2.1 - Non-significant results for main text Table 1.

Variable	Beta	t	p
SFA	-0.0042	-0.0500	0.9604
MUFA	-0.0512	-0.4699	0.6407
PUFA	0.0990	1.4550	0.1526
C8:0	-1.6417	-1.3022	0.2084
C10:0	-0.2358	-0.1513	0.8813
C12:0	0.0607	0.1667	0.8686
C14:0	-0.0774	-0.2978	0.7676
C14:1	-8.3400	-1.9342	0.0681
C15:0	0.5570	0.2174	0.8302
C16:0	0.0225	0.4882	0.6284
C16:1	-0.2201	-0.7229	0.4744
C17:0	0.9517	0.6548	0.5167
C17:1	-0.8565	-0.2655	0.1611
C18:0	0.0251	0.3373	0.7379
tC18:1	1.1994	0.8623	0.3942
cC18:1	-0.0078	-0.0943	0.9254
C18:2(n-6)	-0.0234	-0.5207	0.6086
C18:3	-0.1654	-0.3131	0.7576
C20:0	1.0791	1.1196	0.2703
C20:1	-3.6039	-1.3543	0.1915
C20:3	0.1896	0.9426	0.3577
C22:0	0.2244	0.6389	0.5360
C22:2	0.0587	1.5738	0.1438
C22:6(n-3)	0.2202	0.7677	0.4588
C24:1	0.2672	0.7352	0.4776
Age	0.0000	0.1204	0.9056
Sex	0.0050	0.1341	0.8815
Weight at birth	0.0761	1.6282	0.1219
Birth-to-sampling weight increase/day	0.0811	1.7112	0.1444

2.2 - Non-significant results for Supplementary Table 8.

Weight at birth				Birth-to-sampling weight increase/day			
FA	Beta	t	p	FA	Beta	t	p
C8:0	0.1925	0.1829	0.8568	C8:0	-0.0123	-0.4350	0.6702
C10:0	-0.9266	-0.5912	0.5614	C10:0	-0.0111	-0.2937	0.7733
C12:0	0.1919	0.4296	0.6723	C12:0	-0.0025	-0.2336	0.8187
C14:0	0.2265	0.6605	0.5168	C14:0	0.0045	0.5015	0.6238
C14:1	-0.8964	-0.2664	0.7928	C14:1	-0.1515	-1.5275	0.1489
C15:0	3.3774	1.3626	0.1890	C15:0	0.0358	0.4274	0.6756

C16:0	-0.0635	-0.4398	0.6660	C16:1	0.0167	0.9119	0.3772
C16:1	0.0135	0.0237	0.9813	C17:0	-0.0828	-1.1568	0.2667
C17:0	-3.4064	-1.4131	0.1768	C18:0	-0.0014	-0.7884	0.4407
C17:1	-1.0508	-0.5978	0.5583	tC18:1	0.0047	0.3640	0.7201
C18:0	-0.0603	-0.8020	0.4308	cC18:1	-0.0015	-1.5070	0.1491
tC18:1	0.2966	0.7009	0.4904	C18:2(n-6)	-0.0011	-1.3367	0.1980
cC18:1	0.0061	0.1429	0.8876	C18:3	-0.0058	-0.5567	0.5839
C18:3	0.2836	0.7099	0.4843	C20:0	0.0039	0.0830	0.9349
C20:0	-0.1412	-0.1056	0.9171	C20:1	-0.0156	-0.3792	0.7085
C20:1	-1.1999	-0.6577	0.5167	C20:3	0.0057	1.6396	0.1270
C20:3	0.0213	0.0993	0.9223	C20:4	-0.0113	-1.6413	0.1247
C20:4	0.2248	1.1114	0.2839	C20:5	0.0330	0.6375	0.5349
C20:5	2.3165	1.4099	0.1790	C22:0	-0.0069	-2.0417	0.0570
C22:0	0.0331	0.3174	0.7544	C22:2	0.0066	0.1414	0.8897
C22:2	1.5241	1.0433	0.3133	C22:6(n-3)	0.0250	1.2504	0.2332
C22:6(n-3)	-0.8335	-1.3802	0.1878	C24:1	-0.0466	-0.6990	0.4969
C24:1	-3.1132	-1.4486	0.1680				

2.3 - Non-significant results for Supplementary Table 3. Void entries are significant associations with age.

	Age			Sex		
FA	Beta	t	p	Beta	t	p
C8:0	0.0003	0.6621	0.5139	0.0135	0.2596	0.7973
C10:0	-0.0002	-0.5007	0.6210	-0.0120	-0.3184	0.7528
C12:0	-0.0005	-0.5651	0.5749	0.1930	1.6237	0.1116
C14:0	0.0016	1.1150	0.2709	0.1809	0.9458	0.3494
C14:1	0.0001	0.8972	0.3782	0.0167	1.1019	0.2810
C15:0	0.0001	0.3519	0.7279	-0.0246	-1.1948	0.2434
C16:0	0.0025	0.6247	0.5354	-0.4762	-0.8820	0.3825
C16:1	0.0010	1.3268	0.1914	-0.0480	-0.4740	0.6378
C17:0	0.0004	2.0069	0.0509	-0.0216	-0.8845	0.3812
C17:1				-0.0148	-0.4680	0.6461
C18:0				-0.3877	-0.4805	0.6332
tC18:1	0.0007	0.9926	0.3263	-0.0572	-0.6199	0.5385
cC18:1	0.0162	1.9178	0.0616	0.9956	0.8690	0.3896
C18:2(n-6)	0.0206	1.8838	0.0713	1.1933	0.9298	0.3614
C18:3				0.0194	0.3937	0.6990
C20:0	-0.0003	-1.7593	0.0855	-0.0081	-0.3427	0.7335
C20:1	-0.0003	-1.4807	0.1512	0.0113	0.5343	0.5979
C20:3	-0.0028	-1.1133	0.2762	-0.2145	-0.7163	0.4804
C20:4	0.0011	0.1566	0.8763	-0.2383	-0.2568	0.7985
C20:5	-0.0004	-1.7724	0.0954	0.0063	0.1677	0.8689
C22:0				-0.6730	-1.9656	0.0669
C22:2	-0.0002	-1.0432	0.3124	0.0038	0.1438	0.8875
C22:6(n-3)	-0.0002	-0.3029	0.7659	0.1597	1.5913	0.1311
C24:1				-0.0668	-2.9537	0.3859

2.4 - Non-significant results for main text Table 2.

PD				FD			
Variable	Beta	t	p	Variable	Beta	t	p
SFAs	-0.0088	-0.2792	0.7864	SFAs	0.0599	1.6969	0.1239
MUFAs	0.0175	0.7030	0.4998	MUFAs	-0.0321	-0.7997	0.4445
C18:2(n-6)	0.0136	0.2947	0.7960	C18:2(n-6)	0.0298	1.6037	0.1599
C18:3	2.0590	1.5249	0.2668	C18:3	-0.2205	-0.2873	0.7835
C20:2	-1.4038	-0.9300	0.4505	C20:2	1.4889	1.8629	0.0887
C20:3	-0.0205	-0.0386	0.9727	C20:3	-0.1471	-0.8513	0.4273
C22:4	-1.5001	-1.0892	0.3078	C20:4	-0.0623	-1.7656	0.1079
C22:5	-0.1151	-0.2486	0.8099	C22:4	-0.0623	-1.7656	0.1079
C22:6(n-3)	0.4991	2.2890	0.0513	C22:5	0.1008	0.4118	0.6892
HDL	-1.9040	-0.5259	0.6214	C22:6(n-3)	0.0857	0.4889	0.6354
LDL	-1.8363	-0.5062	0.6342	HDL	-0.1499	-2.5177	0.0864
VLDL	-2.7634	-1.2383	0.2706	LDL	-0.1300	-2.4800	0.0893
Triglycerides	-0.1782	-0.5588	0.6004	VLDL	-2.2190	-2.7252	0.0951
Total cholesterol	1.8483	0.5095	0.6321	Triglycerides	-0.4164	-2.5514	0.1048
Systolic BP	0.0275	2.1369	0.0613	Total cholesterol	0.1292	2.4670	0.0903
Diastolic BP	-0.0178	-0.9547	0.3647	Glucose	0.0372	2.2557	0.1094
Age	0.0141	1.0607	0.3165	Systolic BP	0.0168	1.4624	0.1777
				Diastolic BP	0.0084	0.7887	0.4505
				Age	-0.0001	-0.0077	0.9940

2.5 - Non-significant results for main text Table 3.

PD				FD			
Variable	Beta	t	p	Variable	Beta	t	p
SFAs	0.0312	1.5077	0.1754	SFAs	0.0609	1.6308	0.1470
MUFAs	-0.0109	-0.4218	0.6858	MUFAs	-0.1090	-2.2046	0.0633
PUFAs	0.0018	0.3078	0.7672	PUFAs	-0.0093	-0.4618	0.6582
C18:2(n-6)	-0.0154	-0.5158	0.7118	C18:2(n-6)	-0.0142	-0.4999	0.6611
C18:3	-0.5436	-1.6611	0.2210	C18:3	0.0264	0.2891	0.8912
C20:2	-0.4113	-1.1123	0.2413	C20:2	0.2151	0.1815	0.9002
C20:3	-0.1322	-0.1110	0.4811	C20:3	-0.0959	-0.5114	0.6717
C20:5	-0.1270	-0.4571	0.6585	C20:4	-0.0019	-0.5466	0.6616
C22:4	-0.0974	-0.4997	0.6612	C20:5	0.0458	0.2832	0.8811
C22:5	-0.0662	-0.3113	0.4812	C22:4	0.0298	0.7612	0.5114
C22:6(n-3)	-0.0808	-0.2231	0.7913	C22:5	0.0106	0.4118	0.7713
HDL	1.4501	0.2356	0.8289	C22:6(n-3)	0.0129	1.6114	0.8992
LDL	1.4637	0.2378	0.8273	HDL	-0.1354	-2.1397	0.1219
VLDL	2.1708	0.6644	0.5539	LDL	-0.0935	-1.5133	0.2274
Triglycerides	-0.1418	-0.2432	0.8235	VLDL	-0.4346	-1.3119	0.2809
Total cholesterol	-1.4548	-0.2364	0.8284	Triglycerides	0.0652	1.0303	0.3787

Glucose	-0.0024	-0.2012	0.8534	Total cholesterol	0.0895	1.4498	0.2430
Systolic BP	0.0125	0.9239	0.3863	Systolic BP	0.0011	0.1583	0.8787
Diastolic BP	-0.0010	-0.0558	0.9570	Diastolic BP	-0.0024	-0.3991	0.7017
Age	-0.0012	-0.6370	0.5444	Age	0.0162	1.9307	0.0948
BMI	0.1812	3.3200	0.0671	BMI	0.0025	0.1513	0.8840

2.6 - Non-significant results for Supplementary Table 7.

PD				FD	
FA	Beta	t	p	FA	Beta
C14:0	0.03293	1.02202	0.32872	C14:0	0.01465
C15:0	-0.00639	-0.59916	0.56794	C15:0	-0.00634
C16:0	-0.30933	-1.63823	0.12963	C16:0	-0.1694
C16:1	0.02509	0.61991	0.54795	C16:1	0.00842
C18:0	0.07954	1.07459	0.30556	C20:1	0.01728
C18:3	0.03194	2.16389	0.07368		
C20:1	0.01889	1.66154	0.12481		
C20:3	0.06827	2.15097	0.05455		

2.7 - Non-significant results for Supplementary Table 9.

PD				FD			
FA	Beta	t	p	FA	Beta	t	p
C14:0	2.6749	0.9697	0.40372	C14:0	-15.9718	-2.73499	0.07164
C15:0	-14.79211	-1.46329	0.23958	C15:0	46.2854	1.87408	0.15762
C17:0	-7.10531	-1.31953	0.27866	C16:1	6.69953	2.01807	0.13691
C18:0	0.57799	1.57672	0.21295	C16:0	0.29217	1.33328	0.27464
C18:2(n-6)	6.2486	3.74569	0.16609	C17:0	-8.55814	-1.22366	0.30842
C18:3	-208.46313	-4.42163	0.1416	C18:2(n-6)	-0.21114	-0.21388	0.86586
C20:1	90.64885	5.6082	0.11234	C18:3	-14.4505	-0.35454	0.7831
C20:2	187.86309	4.9139	0.12781	C20:1	-182.90387	-1.51737	0.37096
C20:3	-37.73741	-3.17185	0.19443	C20:2	-0.41912	-0.00735	0.99532
C20:4	-5.8948	-2.26168	0.26503	C20:3	38.24202	2.02865	0.29156
C20:5	2.84867	0.11971	0.92415	C20:4	19.42252	1.90901	0.30719
C22:4	59.85599	2.27185	0.09642	C20:5	83.50241	1.33008	0.41041
C22:6	-7.83781	-0.87636	0.4014	C22:4	-187.10348	-1.90272	0.30805
				C22:5	-38.12658	-1.37374	0.40058
				C22:6	26.69734	1.19489	0.44362

2.8 - Non-significant results for main text Table 4.

PD				FD			
Variable	Beta	t	p	Variable	Beta	t	p
SFAs	0.0766	0.3821	0.7137	SFAs	0.2197	1.5635	0.1619
MUFAs	-0.0689	-0.2751	0.7912	MUFAs	-0.2127	-1.1141	0.3020
PUFAs	0.0760	1.3249	0.2268	C18:2(n-6)	0.3548	0.7875	0.5135

C18:2(n-6)	0.1592	0.1121	0.9289	C18:3	-6.0250	-0.6939	0.5595
C18:3	-10.5092	-0.1328	0.9160	C20:2	4.0409	0.4581	0.6918
C20:2	29.6580	0.2997	0.8146	C20:3	0.3706	0.2163	0.8488
C20:3	-2.0797	-0.1758	0.8892	C22:4	-1.1568	-0.3344	0.7699
C20:4	2.1118	0.8767	0.6964	C22:5	2.5226	0.5047	0.6639
C22:4	22.2071	0.6258	0.6440	C22:6(n-3)	-0.3312	-0.2800	0.8058
C22:5	-33.4401	-1.1094	0.4670	HDL	-0.0941	-0.3018	0.7825
C22:6(n-3)	-2.6068	-0.1542	0.9026	LDL	-0.0982	-0.3228	0.7680
HDL	205.7621	0.4565	0.6928	VLDL	-1.5502	-0.9502	0.4121
LDL	-205.5375	-0.4575	0.6922	Triglycerides	0.2774	0.8897	0.4392
VLDL	-108.1133	-0.4559	0.6932	Total cholesterol	0.0903	0.2972	0.7857
Triglycerides	-19.4755	-0.4565	0.6928	Glucose	0.0705	1.7117	0.1855
Total cholesterol	-205.5949	-0.4574	0.6923	Systolic BP	0.0166	0.7965	0.4519
Glucose	-0.0412	-0.0295	0.9791	Diastolic BP	0.0264	1.4263	0.1968
Systolic BP	0.0432	1.4111	0.2222	Age	0.0429	1.7093	0.1312
Diastolic BP	0.0334	1.3989	0.1994	BMI	0.0289	1.4545	0.1798
Age	0.2011	2.4463	0.0707				
BMI	0.2725	1.5482	0.1965				

Supplementary Table 3 – Significant associations of age at sampling with FAs (dependent variable) adjusted for sex in the LI cohort.

Variable	Beta	t	p	adjusted R^2
C17:1	-0.0004	-2.2275	0.0406	0.1522
C18:0	-0.0150	-2.5320	0.0149	0.0949
C18:3(n-3)	0.0007	2.1569	0.0465	0.1367
C22:0	-0.0082	-3.5946	0.0024	0.4566
C24:1	-0.0006	-3.4719	0.0031	0.4628
C22:0+C24:1	-0.0089	-3.8105	0.0015	0.4912

Beta values indicate the change in percentage FA per one-day age increase. Non-significant associations are shown in Supplementary Table 2.3.

Supplementary Table 4. General characteristics of the AMM subjects.

		BMI Groups		
	All subjects n=12	Normal[b] n=4	Overweight[c] n=4	Obese[d] n=4
Age (years)	34.9 (0.5)	36.5 (2.1)	31.5 (3.9)	36.7 (3.7)
Height (cm)	170.1 (1.8)	166.5 (3.7)	169.0 (2.6)	174.8 (1.8)
Weight (kg)	81.1 (5.2)	61.4 (2.7)**	80.7 (2.6)**	101.4 (4.3)***
BMI (kg/m^2)	28.0 (1.4)	22.4 (0.4)**	28.4 (0.8)*	33.2 (1.5)***
WC (cm)[a]	99.1 (3.7)	85.2 (4.3)*	100.6 (3.7)	111.5 (2.0)**
HC (cm)[a]	106.0 (2.7)	95.8 (1.6)*	105.5 (1.4)**	116.7 (2.1)***
WHR (cm/cm)	0.93 (0.01)	0.88 (0.03)	0.95 (0.02)	0.95 (0.01)
WHtR (cm/cm)	0.58 (0.01)	0.51 (0.02)	0.59 (0.02)	0.63 (0.01)**
SBP (mmHg)	113.3 (3.2)	103.7 (3.7)	112.5 (4.7)	123.7 (3.7)*
DBP (mmHg)	77.9 (1.6)	75.0 (3.5)	78.7 (1.2)	80.0 (3.5)

Data are presented as means (SEM). HC, hip circumference; SBP and DBP, diastolic and systolic blood pressure, respectively; WC, waist circumference; WHR, waist-to-hip ratio; WHtR, waist-to-height ratio. [a] measurements carried out not in the same day as blood extraction; [b] differences between normal and overweight groups; [c] differences between overweight and obese groups; [d] differences between obese and normal groups; *=p<0.05; **=p<0.01; ***=p<0.001; one-way ANOVA.

Supplementary Table 5 - Correlations between metabolic parameters and DNA methylation in subject-averaged repeated measurements (n=5 per subject).

Variable	Day	Pearson's r
TG (mg/dL)	FD	-0.601*
	PD	-0.654*
WBFA (mmol /L)	FD	0.160
	PD	-0.258
HDL (mg/dL)	FD	0.496
	PD	0.129
LDL (mg/dL)	FD	-0.192
	PD	-0.152
VLDL (mg/dL)	FD	-0.600*
	PD	-0.653*
TC (mg/dL)	FD	-0.540
	PD	-0.431
Glucose (mg/dL)	FD	0.077
	PD	-0.521

Supplementary Table 6 - Correlations between metabolic parameters recorded at the second blood draw (12 PM) and DNA methylation recorded at the last draw (6 PM) in subject-averaged repeated measurements (n=5 per subject).

Variable	Day	Pearson's r
TG (mg/dL)	FD	0.080
	PD	-0.057
WBFA (mmol /L)	FD	0.021
	PD	-0.181
HDL (mg/dL)	FD	-0.394
	PD	-0.474
LDL (mg/dL)	FD	0.152
	PD	-0.307
VLDL (mg/dL)	FD	-0.333
	PD	-0.570
TC (mg/dL)	FD	-0.196
	PD	-0.571
Glucose (mg/dL)	FD	-0.722*
	PD	-0.308

Supplementary Table 7 – Significant associations of age with FAs (dependent variable) in the AMM subjects.

Sample	Variable	Beta	t	p	adjusted R^2
PD	C17:0	0.0432	2.3792	0.0365	0.2231
	C20:4	0.2690	3.1271	0.0096	0.3842
	C20:2	0.0311	2.5245	0.0282	0.3042
	C20:5	0.0330	2.5213	0.0284	0.3153
	C22:4	0.1070	2.6456	0.0228	0.2872
	C22:5	0.0713	2.6889	0.0211	0.3105
	C22:6	0.1382	2.9062	0.0143	0.3387
FD	C17:0	0.0344	2.5849	0.0254	0.2674
	C18:0	0.1001	2.3687	0.0372	0.9334
	C18:2	0.4027	2.8105	0.0169	0.6277
	C18:3	0.0259	3.0880	0.0115	0.3921
	C20:2	0.0239	2.6342	0.0232	0.2923
	C20:3	0.0567	2.8687	0.0153	0.6156
	C20:4	0.2529	3.3389	0.0066	0.5474
	C20:5	0.0314	2.9714	0.0127	0.3459
	C22:5	0.0673	2.8145	0.0168	0.3244
	C22:4	0.1043	3.1421	0.0094	0.3787
	C22:6	0.1359	3.3920	0.0060	0.4230

Beta values indicate the change percent FA per one-year age increase. Non-significant associations a
2.6.

Supplementary Table 8 – Significant associations of FAs with weight (dependent variable) in the LI cohort.

	Variable	Beta	t	p	adjusted R^2
Weight at birth	C18:2(n-6)	-0.0725	-2.2201	0.0360	0.1439
Birth-to-sampling weight increase per day	C16:0	-0.0104	-2.2515	0.0409	0.1689
	C17:1	-0.1315	-2.4783	0.0265	

Beta values indicate the change in weight (kg or kg/day) per one-point increase in percent FA. Non-significant associations are shown in Supplementary Table 2.2.

Supplementary Table 9 – Significant associations of FAs with BMI (dependent variable) in the AMM subjects.

Sample	Variable	Beta	t	p	adjusted R^2
PD	C16:0	0.6555	6.0836	0.0089	0.9929
	C16:1	4.2835	4.7498	0.0177	
	C22:5	-77.797	-2.5582	0.0285	
FD	C18:0	2.3419	4.4180	0.0215	0.9704

Beta values indicate the change in BMI per one-point increase in percent FA. Non-significant associations are shown in Supplementary Table 2.7.
